# USP24 stabilizes bromodomain containing proteins to promote lung cancer malignancy

**DOI:** 10.1038/s41598-020-78000-2

**Published:** 2020-11-30

**Authors:** Shao-An Wang, Ming-Jer Young, Wen-Yih Jeng, Chia-Yu Liu, Jan-Jong Hung

**Affiliations:** 1 Department of Biotechnology and Bioindustry Sciences, National Cheng Kung University, Tainan, 701 Taiwan; 2 Graduate Institute of Medical Sciences, College of Medicine, Taipei Medical University, Taipei, Taiwan; 3 School of Respiratory Therapy, College of Medicine, Taipei Medical University, Taipei, Taiwan; 4 University Center for Bioscience and Biotechnology, National Cheng Kung University, Tainan, Taiwan

**Keywords:** Molecular modelling, Chromatin remodelling, Non-small-cell lung cancer, Deubiquitylating enzymes

## Abstract

Bromodomain (BRD)-containing proteins are important for chromatin remodeling to regulate gene expression. In this study, we found that the deubiquitinase USP24 interacted with BRD through its C-terminus increased the levels of most BRD-containing proteins through increasing their protein stability by the removal of ubiquitin from Lys391/Lys400 of the BRD. In addition, we found that USP24 and BRG1 could regulate each other through regulating the protein stability and the transcriptional activity, respectively, of the other, suggesting that the levels of USP24 and BRG1 are regulated to form a positive feedback loop in cancer progression. Loss of the interaction motif of USP24 eliminated the ability of USP24 to stabilize BRD-containing proteins and abolished the effect of USP24 on cancer progression, including its inhibition of cancer cell proliferation and promotion of cancer cell migration, suggesting that the interaction between USP24 and the BRD is important for USP24-mediated effects on cancer progression. The targeting of BRD-containing proteins has been developed as a strategy for cancer therapy. Based on our study, targeting USP24 to inhibit the levels of BRD-containing proteins may inhibit cancer progression.

## Introduction

Deubiquitinases (DUBs) can be divided into the five following subfamilies based on the architecture of their catalytic domains: ubiquitin-specific proteases (USPs), ubiquitin carboxyl-terminal hydrolases (UCHs), ovarian tumor proteases (OTUs), Josephins, and the JAB1/MPN/MOV34 family (JAMM) family^1,2^. Except for the JAMM family, the other DUBs are cysteine proteases, which rely on a catalytic triad of conserved amino acids shared with classical cysteine proteases such as papain^2^. One of cysteine proteases is ubiquitin-specific peptidases 24 (USP24), which is a deubiquitinating enzymes that specifically removes and recognizes ubiquitin from proteins^3^. Malfunctions in USPs are related to many diseases of human, such as cancer progression, inflammation and neurodegenerative disorders^4–6^. Therefore, almost 100 DUBs, which reverse monoubiquitination or polyubiquitination on target proteins to modulate their function and expression have been clarified^7^. Several USPs, such as USP10 and USP24 have been assessed, and their substrates related to cell proliferation and DNA damage repair activity such as p53 have been examined^8,9^. An increasing number of studies have shown that USPs play a pivotal role in controlling the levels of proteins involved in various diseases^10^. Genetic variation in *USP24* was discovered in Parkinson’s disease (PD). Two single nucleotide polymorphisms (SNPs) in USP24 (rs1165222 and rs13312) have been associated with PD risk^11,12^. The proportions of PD and control samples containing another SNP (rs487230) were determined to significantly differ^13^. The detailed mechanisms and functions of USP24 in neurodegenerative diseases remain to be elucidated. In addition, our previous studies indicated that both SNPs are localized within coding region and can affect the mRNA stability, thus affecting USP24 levels. We found that USP24 is highly expressed in more malignant cell lines and clinical samples from late stage lung cancer^14^. Our recent results also indicated that USP24 is downregulated in the early stage of lung cancer, which is advantageous for tumorigenesis^15^. Furthermore, our previous studies found that USP24 is upregulated in late stage lung cancer cells and the tumor-associated microphages around tumors, promoting lung cancer malignancy^16^. Therefore, the DUB USP24 is tightly regulated during lung cancer progression and affects cancer growth and malignancy through stabilizing its substrates by removing the ubiquitin.

Several complexes including the SWI/SNF, ISWI, NuRD/Mi-2/CHD, INO80 and SWR1 complexes have been known to be recruited in genomes to regulate gene expression^17^. One class of proteins, bromodomain (BRD)-containing proteins, which are involved in several chromatin complexes, such as the SWI/SNF and ISWI complexes, regulate gene expression and maintain genomic stability^18^. Regarding gene regulation, these proteins are involved in chromatin remodeling as they regulate gene expression by recruiting other related regulators and further regulate histone modifications, such as acetylation or methylation. For example, PCAF and GCN5, which contain BRDs, act as transcriptional coactivators. Both proteins can interact with other proteins to form the complexes, Spt-Ada-Gcn5-acetyltransferase (SAGA) and ADA2A-containing (ATAC) complexes, in humans to regulate the transcription of many genes^19,20^. Regarding factors that affect genomic stability, including base mutations and genomic structural variants (SVs), are involved in DNA damage repair activity and stabilize genomics SVs^21^. For example, in a recent study, systematic screening of BRD-containing proteins identified homologous recombination and R-loop suppression pathways involved in genomic integrity^22^. BRDs, which are composed of approximately 110 amino acids, can be recruited to acetylated histone H3 and H4 to regulate gene expression^23^. To now, 42 BRD-containing proteins in human have been found, and dysregulation of these proteins, including their mutation or changes in their protein levels, leads to several severe diseases, such as cancer, inflammatory diseases as well as neurodegenerative diseases^24^. While most of these studies have shown that changes in the transcriptional activity of these genes alter their protein levels, few studies about the degradation of these proteins have been reported. A recent study indicated that the E3-ligase FBW7 can increase the polyubiquitination of BRG1, decreasing its protein stability and inhibiting gastric cancer metastasis^16^. No study on the role of DUBs in controlling the levels of BRD-containing proteins have been reported. BRD-containing proteins have been found to be involved in cancer progression. Their aberrant expression can both stimulate and suppress malignancy^25–28^. Most recent studies on these proteins indicate the mechanism by which deregulation of BRD-containing proteins can result in cancer formation, but some other studies have shown that some BRD-containing proteins inhibit tumor formation^29–31^. In this study, we found that USP24 can stabilize BRD-containing proteins through its interaction with the BRD. Therefore, understanding how to control the levels of BRD-containing proteins is critical for controlling gene expression in disease progression.

## Material and methods

### Cell culture and transfection

Human A549 cell line of lung adenocarcinoma epithelial was cultured in RPMI 1640 medium (Invitrogen, Carlsbad, CA, USA), and human H1299 cell line of non-small cell lung carcinoma, and human U2OS cell line of osteosarcoma was cultured in McCoy’s 5A medium (Invitrogen). RPMI and McCoy’s 5A medium contain 100 μg/ml streptomycin sulfate, 10% fetal bovine serum and 100 U/ml penicillin G sodium. All cell lines were kept at 5% CO2 and 37 °C. H1299 and A549 cells were identified and authenticated by Food and Industry Research and Development Institute (Hsinchu, Taiwan). All cell lines were from American Type Culture Collection (ATCC) and were tested for mycoplasma contamination and were negative results. For transfection, cells (3.5 × 10^5^) were seeded on a 6-well plate and were then transfected when they reached 45–55% confluence with plasmids using PolyJet (SignaGen Laboratories, Rockville, MD, USA) or Lipofectamine 2000 (Invitrogen) in accordance with the manufacturer’s instructions^32^.

### Western blotting

Cells were collected by sample buffer and analyzed by electrophoresis. Proteins were transferred to polyvinylidene difluoride (PVDF, Millipore) membrane and TBST buffer (10 mM Tris–HCl, pH 8.0, 150 mM NaCl and 0.05% Tween 20) containing 5% nonfat milk was used for blocking. Anti-USP24 (Cat# 13126-1-AP, Proteintech, 1:3000), anti-ubiquitin (Cat# 9133, Santa Cruz, 1:200), anti-actin (Cat# 110564, Genetex, 1:20,000), anti-myc-tag (Cat# 2272, Cell Signaling, 1:2000), anti-BRG1(Cat# sc-17796, Santa Cruz, 1:1000), anti-BRD7 (Cat# SAB4200047, Sigma-Aldrich, 1:3000), anti-HA (Cat# 12158167001, Roche, 1:3000), anti-GFP (Cat# sc-9996, Santa Cruz, 1:5000), anti-p300 (Cat# 554215, BD, 1:2000), anti-GCN5 (Cat# 114428, Genetex, 1:1000), anti-PB1 (Cat# 100781, Genetex, 1:1000), anti-TIF1α (Cat# 115139, Genetex, 1:1000), anti-Bax (Cat# 50599-2, Proteintech, 1:1000), anti-p53 (Cat# 05-224, Millipore, 1:3000), anti-securin (Cat# ab-79546, Abcam, 1:1000), anti-E2F4 (Cat# ab-150360, Abcam, 1:1000), anti-mdm2 (Cat# sc-13161, Santa Cruz, 1:1000), anti-PCAF (Cat# 109666, Genetex, 1:1000), anti-BRD3 (Cat# 115058, Genetex, 1:1000) were used for probing interested proteins. After incubated with primary antibodies, PVDF membranes were then incubated with secondary immunoglobulin antibodies linked with horse radish peroxidase (Millipore, 1:10,000). ECL Western blotting detection system (Millipore) and ChemiDoc-it imager (UVP) were used for detecting signals.

### Protein stability assay

Cells were infected with scramble or shUSP24 shRNA expressing lentivirus for 4 days, or overexpression of GFP-USP24 or GFP-USP24-CA and treated with 100 μg/ml cycloheximide (Sigma-Aldrich) for different indicated time points to inhibit protein translation. Cells were resolved in sample buffer at indicated time, and protein stability was analyzed by western blot. Protein level was quantified by using Multi Gauge 3.0 software (Fujifilm, Japan).

### Immunoprecipitation

Cell extracts were prepared, and the protein concentration was determined using a bicinchoninic acid (BCA) protein assay kit. Immunoprecipitation was performed as previously described^32^. Briefly, an equal amount of protein was used in each experiment. The supernatants were transferred to new tubes and incubated with anti-HA, USP24, myc or GFP antibodies at a dilution of 1:200 at 4 °C for 12 h. The immunoprecipitated pellets were subsequently incubated with protein G-Sepharose, washed three times with lysis buffer, and separated on SDS-PAGE. After electrophoresis, the gels were processed for immunoblotting with indicated antibodies (1:2000).

### Lentivirus knockdown system

USP24 and scramble knockdown lentivirus were generated from RNAi core facility of Academia Sinica (Taiwan). Cells were maintained in 6-well plates and incubation for 16 h, and then treatment with 1 ml RPMI medium with 10 μg Polybrene (Millipore) and lentivirus with 5 MOI. After infection for 24 h, medium with lentivirus was replaced using fresh medium and kept for another 72 h. The shRNA-expressing lentiviruses targeting USP24 (Clone ID: TRCN0000245779; oligo sequence: 5′-CCGGCTCTCGTATGTAACGTATTTGCTCGAGCAAATACGTTACATACGAGAGTTTTTG-3′; target sequence: 5′-CTCTCGTATGTAACGTATTTG-3′) or scramble (Clone ID: TRCN0000072246; oligo sequence: 5′-CCGGCAAATCACAGAATCGTCGTATCTCGAGATACGACGATTCTGTGATTTGTTTTTG-3′; target sequence: 5′-CAAATCACAGAATCGTCGTAT-3′).

### Wound-healing migration assay

H1299 cells were maintained in 6 cm dishes till 60% density and after overexpression of GFP, GFP-USP24, GFP-USP24-d4-1 or GFP-USP24-C1698A in cells for 24 h, and then cells were scratched with 200 μl tips. Cells were washed with PBS and photographed under microscopy observation. After incubation for another 24 h at 37 °C, migrated distance then was measures and relative migrated distance was analyzed^32^.

### Transwell migration assay

The cell migration assay was performed using Transwell chamber (Costar) with an 8-μM pore size poly carbonate filter membrane. After overexpression of GFP, GFP-USP24, GFP-USP24-d4-1 or GFP-USP24-C1698A in U2OS cells for 24 h, cells were trypsinized and suspended in serum-free McCoy’s 5A. Lower wells were filled with McCoy’s 5A containing 10% fetal bovine serum and upper wells were filled with cell suspensions (2 × 10^4^) in serum-free McCoy’s 5A. After incubation at 37 °C for 6 h, the filter membrane on the lower side was fixation with 10% methanol and stained with DAPI for 3 min. Image of migrated cells were photographed by fluorescence Olympus BX-51 microscopy and migrated cells number were analyzed by ImageJ^32^.

### RT-PCR

Total RNA from cells was extracted by using TRIsure RNA extraction kit (Bioline), and 3 μg of purified RNA was converted into cDNA with SuperScript II reverse transcriptase (Invitrogene) through reverse transcription. PCR was processed with SuperTherm Taq DNA polymerase (GeneCraft) according to manufacturer’s instructions. All values were normalized with internal GAPDH control, and relative levels of gene expression were then calculated. The following primers were used for qPCR: USP24, F:5′-CAGTTGTGCTCTCCTGTGGA-3′, R:5′-AGGGATTTCTCCTGCTCCAT-3′; GAPDH, F:5′-GAGTCAACGGATTTGGTCGT-3′, R:5′-TTGATTTTGGAGGGATCTCG-3′; BRG1, F:5′-AGCGATGACGTCTCTGAGGT-3′, R:5′-GTACAGGGACACCAGCCACT-3′; and BRD7, F:5′-GCTGTTGCACTCAGGAATGA-3′, R:5′-ACTCTTGAAGGCGTGTGCTT-3′.

### Transgenic mice

All experiments and animal care were conducted in accordance with the guidelines and regulations and the experiments related with animals were approved by the Institutional Animal Care and Use Committee (IACUC) at National Cheng Kung University (NCKU), Taiwan (IACUC Approval No. 106223). Transgenic mice were acquired from Jackson Lab (Bar Harbor, MA, USA) and maintained at the National Laboratory Animal Center in Taiwan. Reverse tetracycline trans-activator (rtTA) protein was expressed under the control of Scgb1a1 (secretoglobin, family 1A, member 1) promoter in Scgb1a1-rtTA transgenic mice. To generate the activated Kras (Kras4b^G12D^) in TetO-Kras4b^G12D^ transgenic mice, tetracycline-responsive promoter element (TRE; tetO) was utilized. Kras4b^G12D^ was crossed with Scgb1a1-rtTA transgenic mice to generate bitransgenic mice. In order to induce the generation of Kras4b^G12D^ in bitransgenic mice, doxycycline (0.5 g/l) was added to the drinking water, starting at the age of 6 weeks. Transgenic mice were performed as previously described^32^.

### Collection of specimens from lung cancer patients

All human study has been conducted in accordance with the guidelines and regulations. The study using human specimens was approved by the Clinical Research Ethics Committee at National Cheng Kung University Medical Center (Tainan, Taiwan). After surgical resection at National Cheng Kung University Hospital, specimens of patients with lung adenocarcinomas were collected for Immunohistochemical analysis or western blotting. The pathological data were analyzed by clinical pathologists. Informed consent was obtained from all subjects. Collection of specimens from lung cancer patients was performed as previously described^32^.

### Immunohistochemistry

Human specimens were fixed in 10% formaldehyde for 72 h for dehydration. For IHC, paraffin-embedded sections were dewaxed by xylene (Sigma-Aldrich) and dehydration by serial diluted ethanol. Incubation in PBS containing 0.3% hydrogen peroxide (Sigma-Aldrich) blocked endogenous peroxidases for 30 min, and then 1% bovine serum albumin blocked samples. Incubation with anti-USP24 (Cat#13126-1-AP, Proteintech, 1:200) and anti-BRD7 (Cat# SAB4200047, Sigma-Aldrich, 1:200) recognized proteins of interest for 3 h at room temperature and using Vectastain ABC kit (Vector) detected immunoreactivity. Sections were photographed by Olympus BX-51 microscope.

### Structural modeling

In order to interpret putative interactions between human USP24 and BRD2 from a structural point of view, we created a 3D structural model of the catalytic domain of human USP24 (USP24-CD) in complex with the second bromodomain of human BRD2 (BRD2-BD2). The structural model of USP24-CD was generated by the SWISS-MODEL server^33,34^, a fully automated protein structure homology-modelling server, using the Protein Data Bank (PDB) code 5KYC (the catalytic domain of human USP7) as the template. The complex structural model of human USP24-CD interacting with BRD2-BD2 was generated using the ZDOCK server^35,36^, a website that performs a full rigid-body search of docking orientations between two proteins, using the modeling structure of USP24-CD and crystal structure of BRD2-BD2 (PDB code 4MR5) as the input models. The top one docking model of USP24-CD/BRD2-BD2 complex was selected to produce the structural figure using PyMOL (http://www.pymol.org).

### Statistical analysis

The comparison between two groups was performed using Student’s t-test. A *p* value < 0.05 was considered to represent a significant difference.

## Results

### USP24 stabilizes BRD-containing proteins

**Figure 1:**
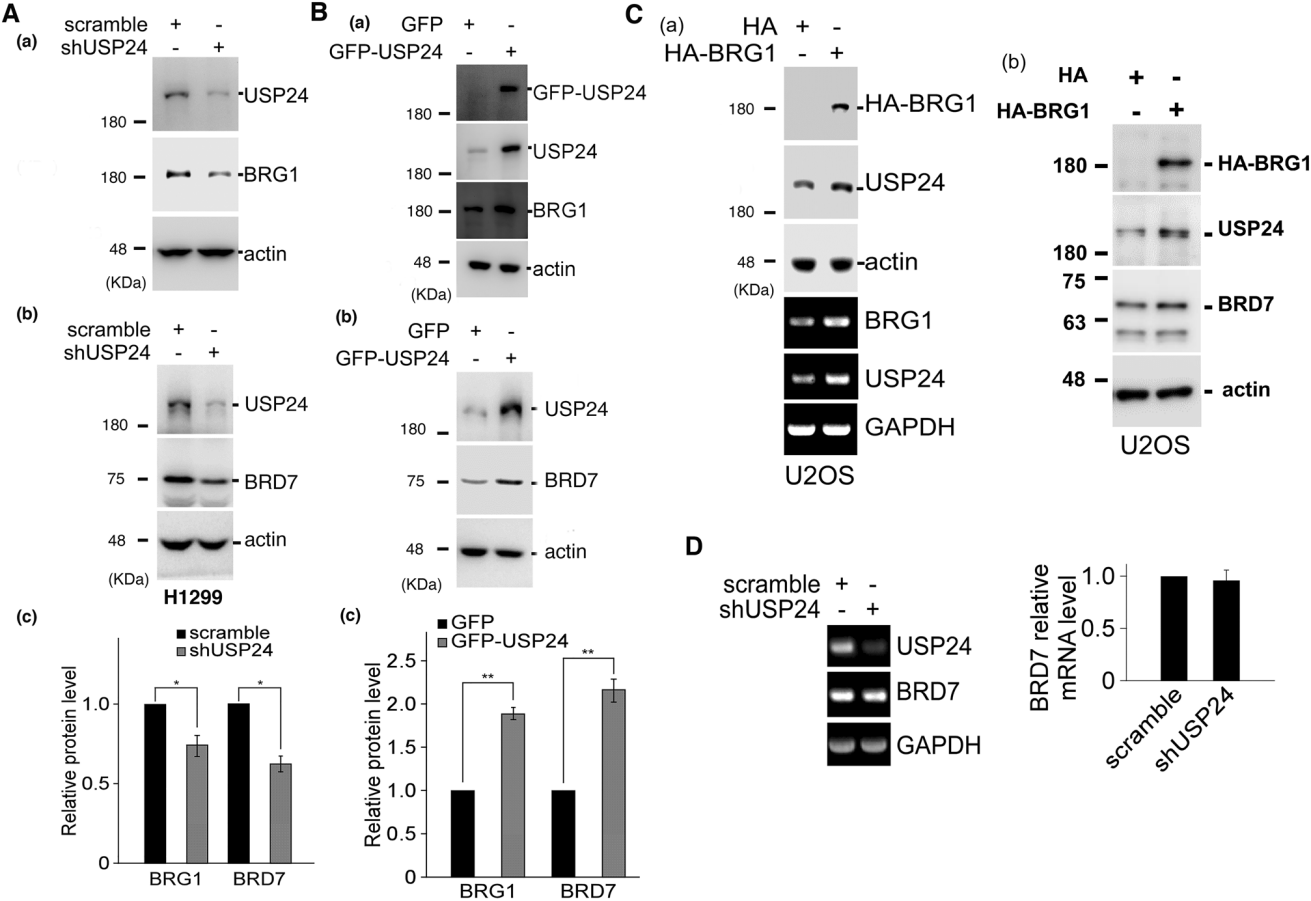
USP24 increases BRD-containing protein expression. Samples were harvested from H1299 lung cancer cells or U2OS bone cancer cells with or without knockdown of USP24 by shRNA (**A**) or with or without overexpression of GFP-USP24 (**B**). The levels of USP24, BRG1 and BRD7 were determined by Western blotting with the indicated antibodies. The relative indicated protein levels of the indicated genes were quantitated, and the results underwent statistical analysis by t-test; *p < 0.05; **p < 0.01, after three independent experiments. The effects of overexpression of HA-BRG1 on the protein and mRNA levels of BRG1, USP24 and BRD7 in U2OS cells were studied by Western blotting and RT-PCR, respectively (**C**). The mRNA levels of USP24 and BRD7 were studied by RT-PCR (**D**). The relative BRD7 mRNA levels were quantitated compared to control after three independent experiments.

**Figure 2:**
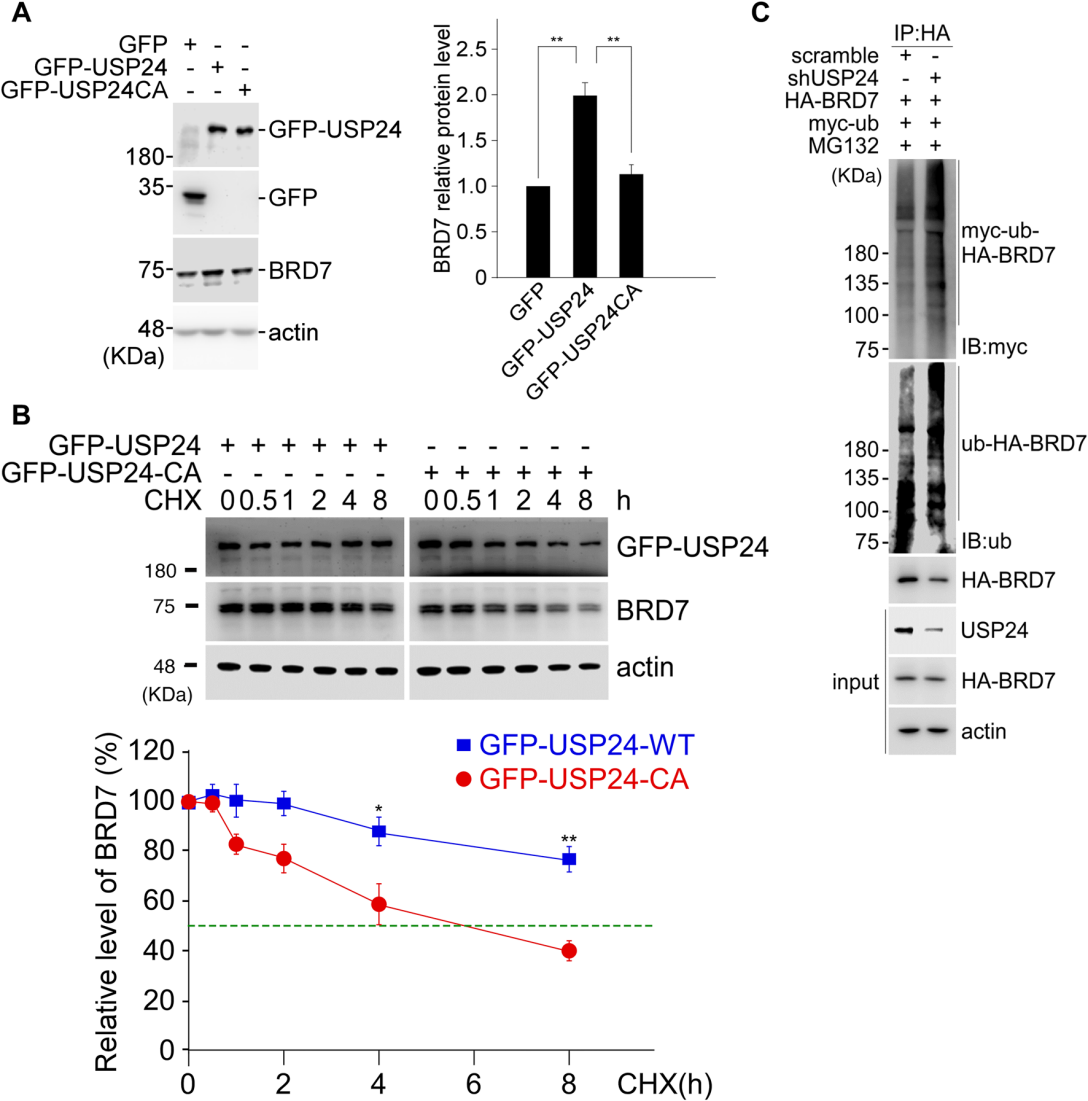
USP24, a deubiquitinase, stabilizes BRD7. The levels of GFP-USP24 or GFP-USP24CA in H1299 lung cancer cells after overexpression of GFP-USP24 or GFP-USP24CA were determined (**A**), and the protein stability of BRD7 in the presence of cycloheximide was determined by Western blotting with the indicated antibodies, BRD7 was quantitated and the results underwent statistical analysis by t-test, *p < 0.05; **p < 0.01, after three independent experiments (**B**). The ubiquitin signal was determined by using IP with anti-HA antibodies and then Western blotting with anti-ubiquitin and anti-myc antibodies (**C**).

When we recently used the C-terminus of USP24 as a bait to probe USP24-interacting proteins in a yeast two-hybrid assay, more than one hundred proteins were recruited by the C-terminal end of USP24 (a.a. 2078-2620). Several BRD-containing proteins, including BRD2, BRD7 and BRDT, could be recruited by USP24 (Suppl. Fig. 1A). Our previous results also indicated that USP24 can interact with p300, an acetyltransferase that contains a BRD^15,32^. These results imply that most or all BRD-containing proteins can interact with USP24. Next, we aimed to determine the role of the interaction between USP24 and BRD-containing proteins. USP24 knockdown (KD) in H1299 lung cancer cells and U2OS bone cancer cells decreased levels of the BRD-containing proteins including BRG1, BRD7, BRD1, BRD3, GCN5, PCAF, and TIF1α (Fig. 1A and Suppl.Fig. 1B). Overexpression of GFP-USP24 in H1299 cells increased BRG1 and BRD7 protein levels but did not affect their mRNA levels, indicating that USP24, a DUB, may stabilize protein stability instead of increasing transcriptional activity (Fig. 1B,D). Since BRG1 is a major member of the chromatin remodeling complex, which regulates the expression of many genes, overexpression of HA-BRG1 increased the protein and mRNA levels of USP24, suggesting that USP24 and BRG1 can regulate each other through modulating the protein stability and transcriptional activity, respectively, of the other, which may be critical for cancer progression (Fig. 1C(a)). In addition, overexpression of BRG1 also increased the level of BRD7 (Fig. 1C(b)). To study the catalytic importance of USP24, GFP-USP24-WT and GFP-USP24-CA, an enzymatically dead form of USP24, were transfected into cells, and the level of BRD7 was determined (Fig. 2A). The data indicated that USP24 increased the level of BRD7, but GFP-USP24-CA can’t, implying that the catalytic activity of USP24 is essential for increasing BRD7 protein levels. Next, we studied the protein stability of BRD7 in the overexpression of GFP-USP24-WT or GFP-USP24-CA with cycloheximide treatment for different time course in H1299 lung cancer cells (Fig. 2B). Overexpression of GFP-USP24-WT increased the protein stability of BRD7, but not under overexpression of GFP-USP24-CA. In addition, knockdown of USP24 and overexpression of GFP-USP24 in U2OS bone cancer cells decreased and increased the protein stability of BRD7, respectively (Suppl. Fig. 2). Since USP24 is a DUB, the ubiquitination of BRD7 was studied under USP24-KD conditions (Fig. 2C). The data indicated that the loss of USP24 increased the signal for HA-BRD7 ubiquitination, suggesting that USP24 can remove ubiquitin from ubiquitinated BRD7 to increase its stability, thereby increasing its level.

### The BRD is a substrate of USP24

**Figure 3:**
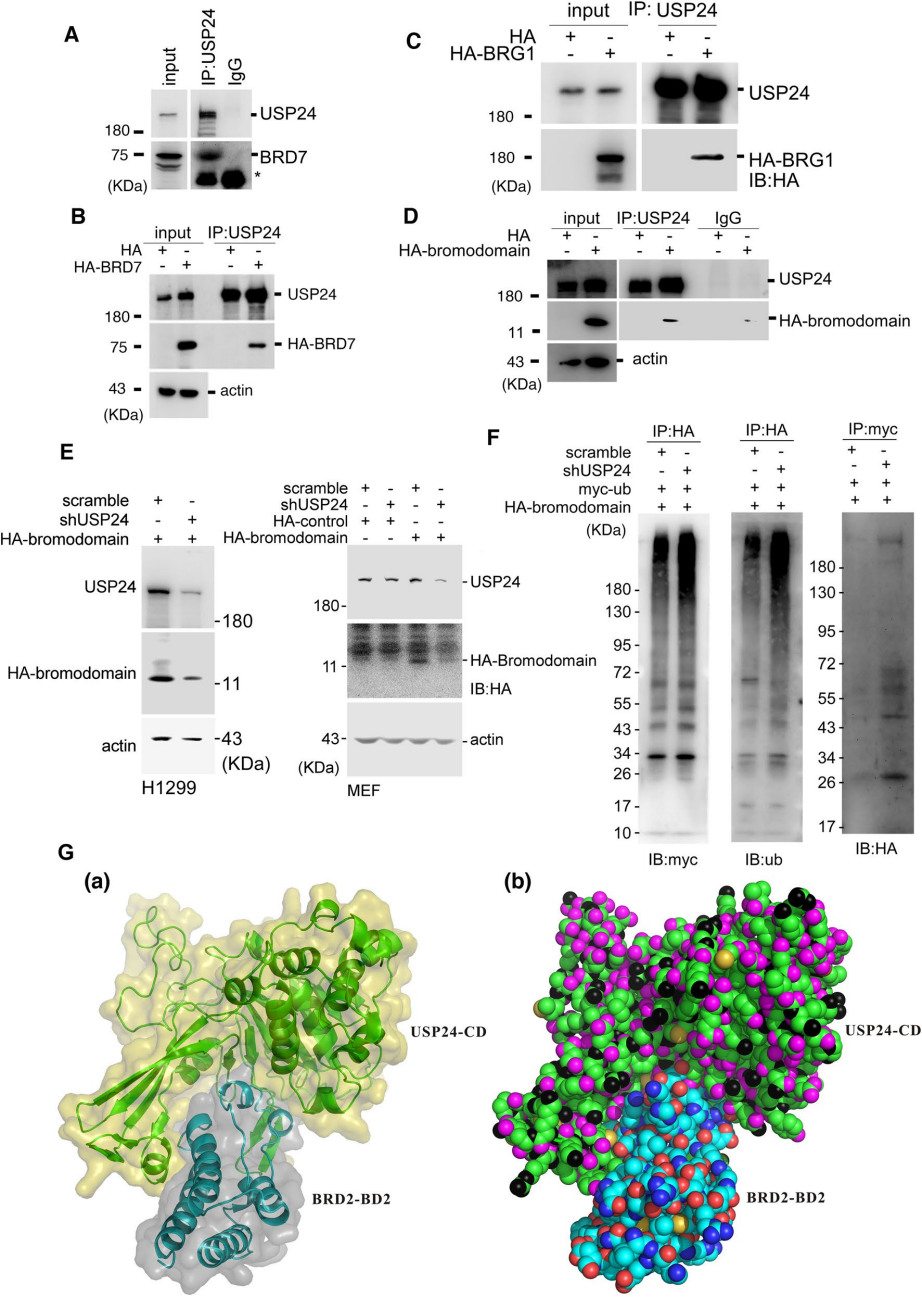
USP24 interacts with the BRD and stabilizes its protein stability. The H1299 cell lysate was used for IP with anti-USP24 antibody, followed by Western blotting with anti-USP24 and anti-BRD7 antibodies (**A**). HA-BRD7 (**B**) or HA-BRG1 (**C**) was expressed in H1299 cells that were subjected to IP with anti-USP24 antibody, followed by Western blotting with anti-HA and anti-USP24 antibodies. HA-BRD was expressed in H1299 cells that were subjected to IP with anti-USP24 and IgG antibodies; followed by Western blotting with anti-USP24 and anti-HA antibodies (**D**). H1299 cells and MEFs cells overexpressing myc-Ub and HA-BRD and those in which USP24 had been knocked down were subjected to Western blotting with the indicated antibodies (**E**). The ubiquitination signal for HA-BRD in H1299 cells following overexpression of myc-Ub and HA-BRD and knockdown of USP24 was determined by IP with anti-HA or anti-myc antibodies, following which Western blotting was carried out with indicated antibodies (**F**). The interaction between USP24 and the BRD was modeled (**G**). A structural model of the complex between the catalytic domain of human USP24 (USP24-CD) and the second BRD of human BRD2 (BRD2-BD2) is shown. (**G**(a)) The USP24-CD molecule is shown as an olive surface and a green cartoon diagram. The BRD2-BD2 molecule is shown as a gray surface and a cyan cartoon diagram. (**G**(b)) The USP24-CD and BRD-BD2 molecules are shown as spheres. The carbon, nitrogen and oxygen atoms of the USP24-CD molecule are shown in green, black, and magenta, respectively. The carbon atoms, nitrogen atoms and oxygen atoms of the BRD2-BD2 molecule are shown in cyan, blue and red, respectively. Sulfur atoms are shown in gold.

**Figure 4:**
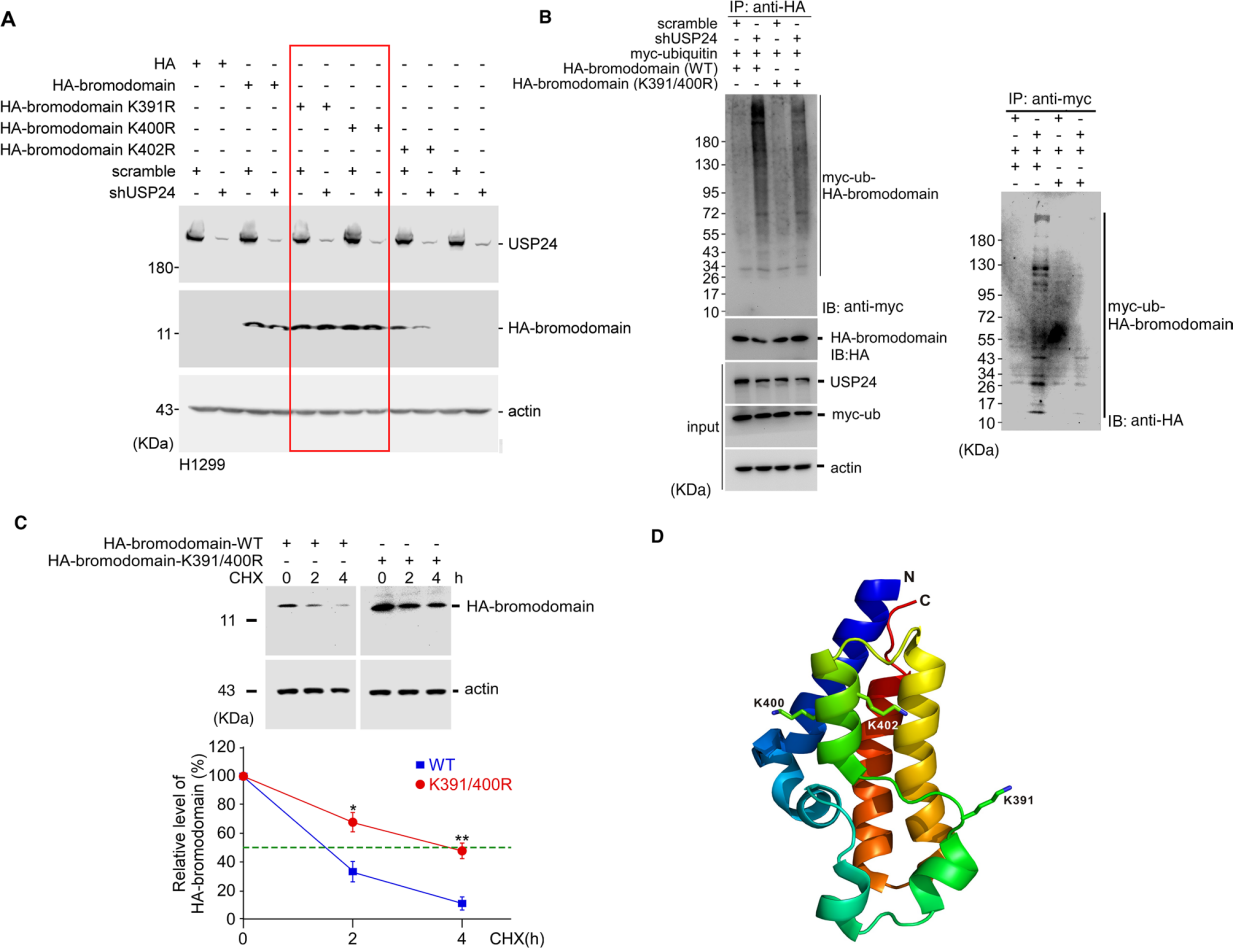
USP24 removes ubiquitin from Lys391 and Lys400 of the BRD. HA-BRD and its various HA-BRD mutants were expressed in USP24-silenced H1299 cells, following which Western blotting was performed with anti-USP24, anti-HA and anti-actin antibodies (**A**). The HA-BRD and its mutant, HA-BRD (K391/400R) were expressed in USP24-silenced H1299 cells for IP with anti-HA and anti-myc, and then Western blot with indicated antibodies (**B**). HA-BRD and the mutant HA-BRD (K391/400R), were expressed inside H1299 cells in the present of cycloheximide, and lysates were collected at the indicated time for Western blotting with anti-HA antibody, and The BRD and mutant BRD were quantitated, and the results were subjected to statistical analysis by t-test, *p < 0.05; **p < 0.01, after three independent experiments (**C**). The localization of Lys391 and Lys400 in the structure of the BRD are shown (**D**).

Since we found that several BRD-containing proteins could be recruited by USP24 in yeast two-hybrid assay and USP24 can increase the level of BRD7 through increasing in its protein stability, USP24 may stabilize BRD-containing proteins. We further studied the molecular mechanism by which USP24 stabilizes BRD-containing proteins (Fig. 3). First, we found that endogenous USP24 could interact with BRD7 in H1299 cells and mouse embryonic fibroblasts (MEFs) (Fig. 3A and Suppl.Fig. 3A). Next, when HA-BRD7 and HA-BRG1 were expressed in H1299 cells or MEFs, we found that USP24 interacted with BRD7 and BRG1, indicating that USP24 could interact with more than one BRD-containing protein in different cell lines (Fig. 3B,C and Suppl.Fig. 3B). Because USP24 could interact with more than one BRD-containing protein, we hypothesized that USP24 can interact with the BRD, which is common to all BRD-containing proteins. Therefore, we constructed HA-BRD, expressed it in the cells, and studied its interaction with USP24 (Fig. 3D). The data indicated that USP24 could indeed interact with HA-BRD, implying that all BRD-containing proteins can interact with USP24 to regulate their protein stability. Next, we studied the role of USP24 in regulating HA-BRD levels in H1299 cells and MEFs (Fig. 3E). Knockdown of USP24 significantly decreased the level of HA-BRD in H1299 cells and MEFs. Moreover, the level of HA-BRD in USP24-KD H1299 cells was rescued by MG132 treatment (Suppl.Fig. 4). Furthermore, the signal of HA-BRD ubiquitination was assessed in H1299 lung cancer cells (Fig. 3F). All data suggested that the loss of USP24 increased the ubiquitination of HA-BRD, indicating that USP24 interacts with BRD-containing proteins at their BRD and removes the ubiquitin molecule(s) localized at the BRD, stabilizing the protein stability of BRD-containing proteins. Finally, we also used a structural model of USP24 built based on USP7 as a parent template to study the structural interaction between USP24 and the BRD (Fig. 3G). Owing to the absence of structural information on the interaction between USP24 and the BRD, we generated a structural model of the catalytic domain of human USP24 (USP24-CD) by using the SWISS-MODEL server, and a complex structural model of the interaction between USP24-CD and the second BRD of human BRD2 (BRD2-BD2) was built up by using the ZDOCK server, which provided a structural view to understand the interactions between USP24-CD and BRD2-BD2 (Fig. 3G(a)). The USP24-CD/BRD2-BD2 complex model revealed that the substrate-binding cavity of USP24-CD can interact well with BRD2-BD2 (Fig. 3G(b)).

Furthermore, we investigated the ubiquitination site(s) within the BRD (Fig. 4 and Suppl. Fig. 5). Twelve lysine residues were found in the BRD. Therefore, it was difficult to determine the exact ubiquitinated residue(s) by their individual mutation. Therefore, we first collected many BRD sequences from various BRD-containing proteins for a sequence alignment and found that three lysine residues, Lys391, Lys400 and Lys402, are highly conserved in these BRD-containing proteins and thus might be critical (Suppl. Fig. 5). Therefore, we individually mutated these three lysine residues to arginine residues and then transfected the plasmid into cells to study their levels with or without USP24 KD (Fig. 4A). The data indicated that the mutation of Lys391 and Lys400, but not Lys402, rescued the BRD level. Next, Lys391 and Lys400 were mutated together, and the plasmid was transfected into cells to study the signal of HA-BRD ubiquitination and the HA-BRD protein stability (Fig. 4B,C). The data indicated that the ubiquitination signal of HA-BRD was decreased in HA-BRD (K391/400R)-expressing cells compared to wild-type HA-BRD-expressing cells, indicating that these two lysine residues indeed partially contributed to ubiquitination of the BRD. In addition, when we studied the protein stability, we found that the stability of HA-BRD (K391/400R) was increased compared to that of wild-type HA-BRD (Fig, 4C). Finally, we visualized these two lysine residues in the BRD structure and found that both lysine residues are localized on the surface of the structure, which may be beneficial for the processing of related enzymes such as USP24 and E3 ligases (Fig. 4D).

**Figure 5:**
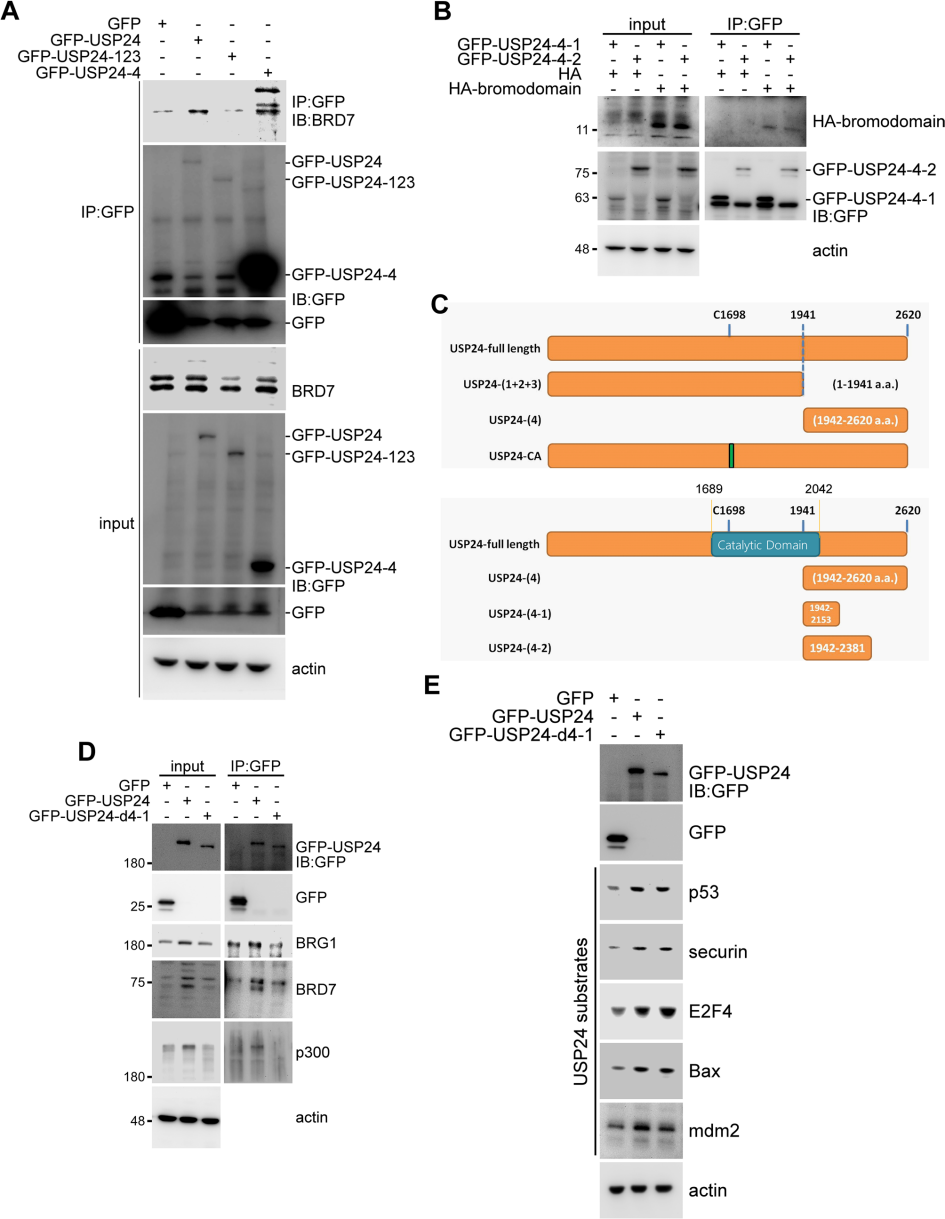
BRD interacts with the C-terminus of USP24. GFP-USP24 and truncated forms of GFP-USP24 were expressed in cells for IP with anti-GFP antibodies, followed by Western blotting with the indicated antibodies (**A**,**B**). A schematic diagram of the GFP-USP24 and truncated forms of GFP-USP24 (**C**). GFP-USP24 and the mutant, GFP-USP24-d4-1, were expressed in cells for IP with anti-GFP antibody to study the interaction between the indicated proteins (**D**) and levels (**E**) of the indicated proteins with Western blotting with indicated antibodies.

### BRD interacts with the C-terminus of USP24

**Figure 6:**
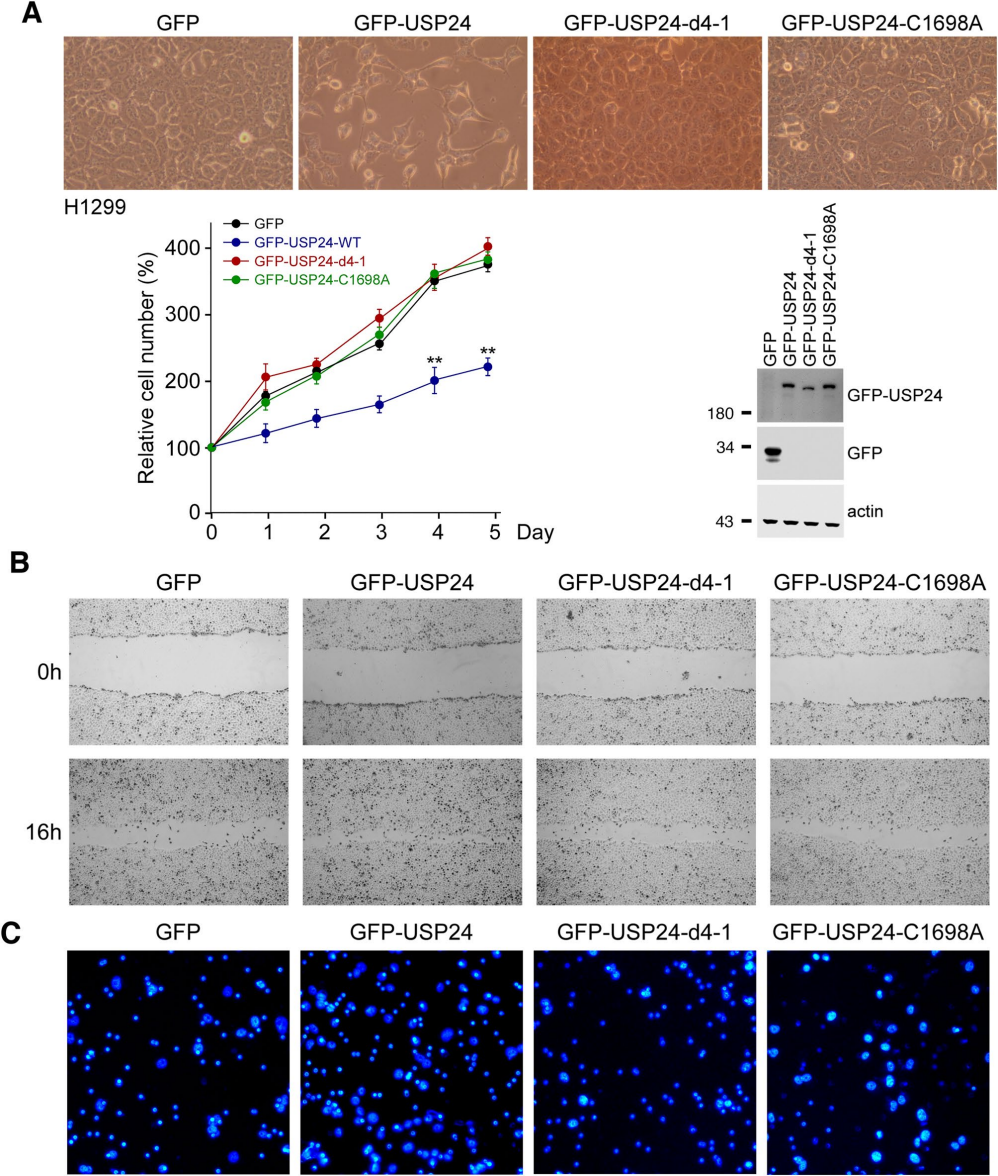
Loss of the BRD-interacting region of USP24 abolished the effect of USP24 on lung cancer progression, including proliferation and metastasis. GFP-USP24 and GFP-USP24 mutants were expressed inside cells to study the cell proliferation by counting the cell numbers, and the cells were quantified and statistical analysis was performed by t-test, **p < 0.01, after three independent experiments (**A**). GFP-USP24 and its mutants were expressed in H1299 cells, and their effects on cell migration by wound healing (**B**) and transwell migration assays (**C**).

At this point, we had delineated that the protein stability of most BRD-containing proteins can be regulated by USP24 through its interaction with the BRD and removal of the ubiquitin from the BRD. Because BRD-containing proteins are crucial for controlling genomic instability and gene expression, understanding the regulation of BRD-containing proteins is critical. To address the importance of the interaction between USP24 and BRD-containing proteins in cancer progression, we needed to abolish the interaction and study the effects on cancer progression. First, we needed to identify the interaction motif(s) between USP24 and the BRD (Fig. 5). Based on the results of a yeast two-hybrid assay, the interaction region of USP24 is localized in its C-terminus. Therefore, we first constructed an N-terminal USP24 construct, USP24-(1-2–3) and a C-terminal USP24 construct, USP24-(4), to study the interaction of USP24 with BRD7 (Fig. 5A,C). The data indicated that BRD7 interacted with full-length USP24 and the C-terminus of USP24, USP24-(4), but not USP24-(1-2-3), which is consistent with the results of the yeast two-hybrid assay. Furthermore, we tried to narrow down the interaction motif to either USP24-(4-1) or USP24-(4-2) to study the interaction of USP24 with HA-BRD (Fig. 5B,C). The data indicated that USP24-(4-1) (a.a.1942-a.a.2153) was sufficient for the interaction between USP24 and BRD (Fig. 5B). Next, a USP24 mutant, GFP-USP24-d4-1, in which this interaction motif was absent, was constructed to study the role of the interaction between USP24 and BRD-containing proteins (Fig. 5D,E). GFP-USP24 increased the levels of not only the BRD-containing proteins BRG1, BRD7, p300, GCN5, PB1 and TIFα but also the other USP24 substrates p53, securin, E2F4, Bax and mdm2. However, with the loss of the interaction motif, GFP-USP24-(4-1), still stabilized other USP24 substrates but not BRD-containing proteins. All these data indicated that loss of the interaction motif of USP24 with the BRD abolished the effect of USP24 on BRD-containing proteins but did not affect the catalytic activity of USP24 for other substrates. We continued to use this construct to study the role of the interaction between USP24 and BRD-containing proteins in lung cancer progression (Fig. 6). Overexpression of GFP-USP24-WT, but not GFP-USP24-d4-1 or GFP-USP24-C1698A, inhibited cell proliferation, indicating that the interaction between USP24 and BRD-containing proteins is critical for USP24-mediated inhibition of cell proliferation (Fig. 6A). Finally, we also studied the effect of the interaction between USP24 and BRD-containing proteins on the migratory ability of lung cancer and bone cancer cells (Fig. 6B,C and Suppl.Fig. 6). GFP-USP24-WT, but not GFP-USP24-d4-1 and GFP-USP24-C1698A, significantly increased the migratory abilities of lung cancer and bone cancer cells. All these results indicated that regulation of the levels of BRD-containing proteins by USP24 is important for the function of USP24 in cancer progression, including its inhibition of cellular proliferation and increase in cancer migratory ability in lung cancer and bone cancer cells.

### USP24 and BRD-containing proteins are upregulated in a lung cancer animal model and clinical cohorts

**Figure 7:**
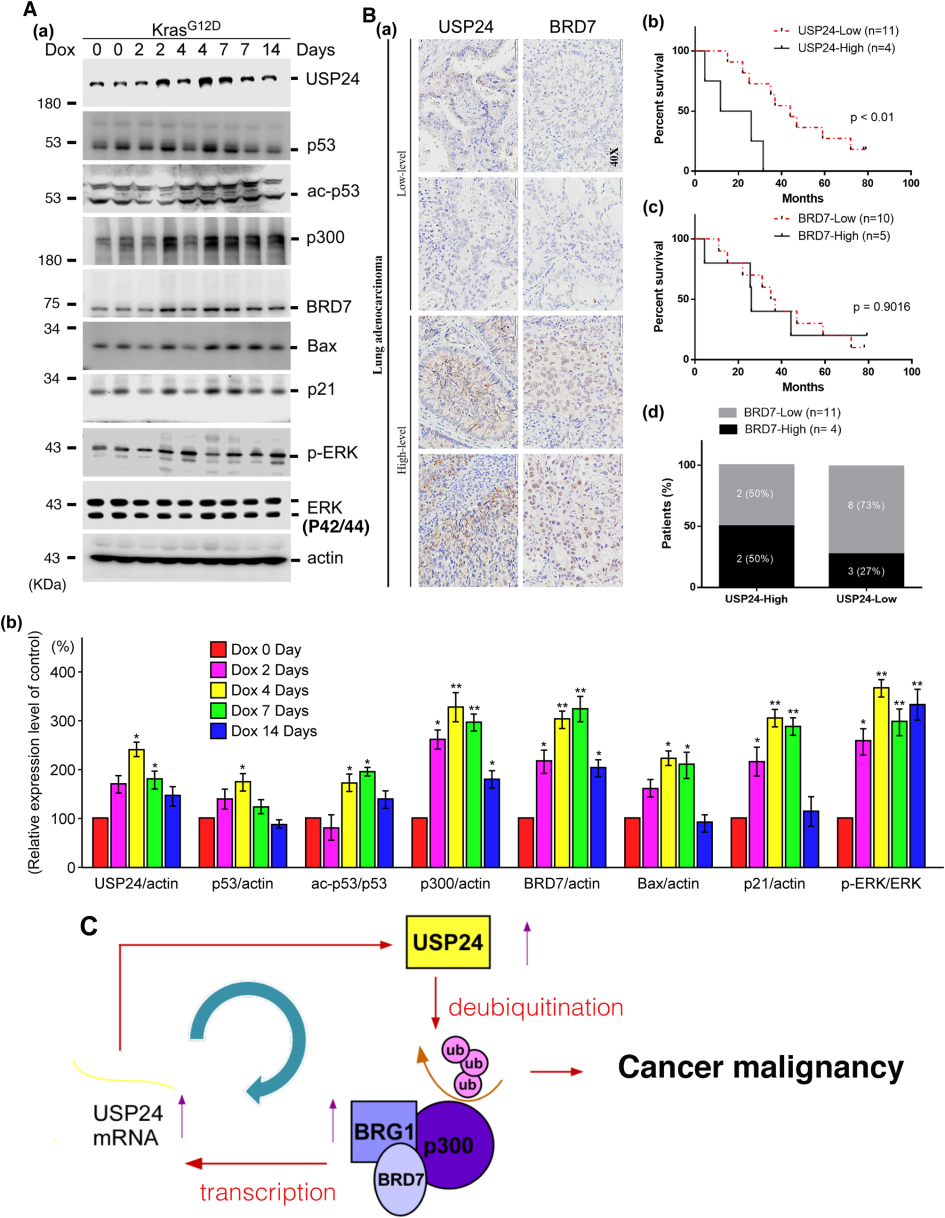
The correlation between USP24, BRD7 and related proteins in Kras^G12D^-induced lung cancer mice and clinical cohorts. Lung tumors were obtained from Kras^G12D^ mice treated with doxycycline, sacrificed at the indicated times to study the related protein levels by Western blotting with the indicated antibodies. There are 5 mice in each group. (**A**(a)), the indicated proteins were quantified compared to control and statistical analysis was performed by t-test, *p < 0.05; **p < 0.01, after 5 independent experiments (**A**(b)). The levels of USP24 and BRD7 in 15 clinical lung cancer patients were determined by IHC with anti-USP24 and anti-BRD7 antibodies (**B**(a)). The relevance between survival rate and levels of USP24 (**B**(b)) and BRD7(**B**(c)), and the correlation between USP24 and BRD7 (**B**(d)) were determined. A working model: USP24 and BRG1 regulate each other, and the interaction regulates lung cancer progression (**C**).

Since BRD-containing proteins are not only related to cellular progression but also involved in cancer initiation^37,38^. The levels of USP24 and its substrates in the initiation of lung cancer were determined in Kras^G12D^-induced lung cancer mice (Fig. 7A). Interestingly, after doxycycline treatment for 2–7 days, the levels of USP24 and its related substrates p53, acetyl p53, p300, BRD7, Bax, and p21 were increased (Fig. 7A), which may be related to cancer initiation. In addition, we used 15 lung cancer clinical specimens to study the correlation between the levels of USP24 and BRD7 (Fig. 7B(a,d)). Patients with higher USP24 levels also had higher BRD7 levels, implying that USP24 really regulates most BRD-containing proteins under physiological and pathological conditions. Finally, the relevance between survival rate and levels of USP24 and BRD7 was studied (Fig. 7B(b,c)). Data indicated that higher USP24 level led poor prognosis significantly, but only slightly relevance in BRD7 level in lung cancer progression.

## Discussion

In this study, we found that USP24 and BRG1 regulate each other and that this regulation is beneficial for cancer progression. USP24 can stabilize BRG1, and BRG1 increases the transcriptional activity of USP24, thus modulating cancer progression by both inhibiting proliferation and promoting migratory ability of cancer cells (Fig. 7C).

More than one hundred of DUBs in human cells are responsible for regulating the levels of most of proteins through deubiquitination^39^. Dysregulation of these DUBs results in the loss of control of protein levels, leading to various diseases, including cancer and neurodegenerative diseases^40^. Several novel inhibitors have been developed and show potential for clinical use in the future^41,42^. Our previous studies revealed that USP24 inhibits cell proliferation through affecting the EGF-activating pathway, p300 and Bax-mediated apoptosis, and securin-regulated anaphase^15^. In this study, USP24 was shown to interact with the BRD to stabilize most BRD-containing proteins, including p300. Previous studies indicated that p300 can acetylate p53 to activate its activity, leading to cell apoptosis, which is consistent with the findings of this study^43^. In addition, p53 has been shown to be a substrate of USP24^9^. However, our study indicates that loss of the interaction motif of USP24 for the BRD still increased the level of p53, indicating that the loss of this motif affects only BRD-containing proteins but not on other substrates.

In this study, we also found that loss of the interaction between USP24 and BRD-containing proteins abolished the effect of USP24 in promoting cancer migratory ability, indicating that USP24-stabilized BRD-containing proteins are important for enhancing cancer malignancy. Our previous studies also indicated that USP24 upregulation in lung cancer cells and the tumor-associated macrophages enhanced cancer malignancy through increases in IL6 expression and secretion via p300-mediated acetylation of histone H3^16^. The other study also indicates that USP24 increased Suv39h1 to promote cancer malignancy^14^. Many previous studies have reported that BRD-containing proteins are important for genomic instability, gene regulation and chromatin remodeling by their formation of complexes and recruitment to acetyl histones^22,44^, which are physiologically and pathologically critical. BRD-containing proteins are important members of chromatin remodeling complexes, such as the SWI/SNF complex, which can control gene expression by controlling compaction and decompaction of the genome^45^. The SWI/SNF complex is responsible for ATP-dependent control of chromatin structure, which stimulates transcription^46,47^. BRG1, one of the core subunits of the SWI/SNF complex, has ATPase activity to effectuate ATP-dependent recruitment to acetylated histones H3 and H4 by the BRD^48^. In this study, we found that USP24 and BRG1 regulate each other and that this regulation is important for cancer metastasis. Our previous studies indicated that USP24 positively regulates lung cancer metastasis^14,16^. In addition, increasing evidence shows that BRG1 also promotes cancer metastasis^49–52^. For example, a recent study indicated that FBW7-mediated degradation of BRG1 inhibits gastric cancer metastasis, indicating that BRG1 is a positive regulator of cancer malignancy^16^. A recent study also indicated that targeting BRG1 is an effective approach in the treatment of PTEN-deficient prostate carcinoma (PCa)^38^. Taken together, these results indeed indicate that USP24-mediated stabilization of BRD-containing proteins is critical for cancer progression^14,16^. BRD7, another important BRD-containing protein, is involved in the expression of genes, such as Nodal, ADAMTs, BMI1 and CRABP1 as well as the regulation of thyroid-releasing hormone^53^. Although several papers have shown that BRD7 is a tumor suppressor, a previous study indicated that miRNA-410 increased cell proliferation in non-small cell lung cancer by targeting BRD7, but the role of BRD7 in the metastasis of lung cancer remains unknown, although BRD7 can inhibit the proliferation of lung cancer cells^54^. Herein we found that USP24-mediated stabilization of BRD-containing proteins inhibited lung cancer cell growth but also increased the migratory ability of lung cancer cells, which is consistent with the effect of USP24 on lung cancer progression^14–16^. In addition, numerous studies have indicated that BRD-containing proteins are involved in regulating genomics stability^22^. USP24-stabilized BRD-containing proteins may also be involved in genomic stability and thus contribute to heterogeneity during cancer progression. Therefore, the future development of novel inhibitor(s) of USP24 to inhibit cancer malignancy shows potential.

Regarding the regulation of BRD-containing proteins in cancer progression, in this study, we not only found that most BRD-containing proteins are substrates of USP24 but also elucidated the interaction motif of USP24 and the residues that are ubiquitinated in the BRD, Lys391/Lys400. Since all 42 members of the BRD-containing proteins family are very important for gene regulation under various physiological and pathological conditions, many studies regarding mechanisms to regulate their function, including maintaining their levels, posttranslational modifications (PTMs) and complex formation, have been carried out. This is the first study to show that the DUB USP24 directly deubiquitinates the BRD to all BRD-containing proteins. Several inhibitors of BRD-containing proteins, such as BETs, have been developed to inhibit cancer progression^55^. In this study, we found that USP24 and BRG1 can regulate each other. Drug targeting USP24 to inhibit cancer malignancy may have potential for clinical use as anticancer drugs in the future.

## Supplementary information

Supplementary Figures.
