# Supplementary material for: USP24 stabilizes bromodomain containing proteins to promote lung cancer malignancy
Source: Sci Rep. 2020 Nov 30;10:20870. doi: 10.1038/s41598-020-78000-2 (PMC7705756; doi:10.1038/s41598-020-78000-2)
Supplement: Supplementary file 1 — Supplementary Figures. [file 41598_2020_78000_MOESM1_ESM.pdf]

USP24 Stabilizes Bromodomain Containing Proteins to Promote Lung Cancer Malignancy

Shao-An Wang, Ming-Jer Young, Wen-Yih Jeng, Chia-Yu Lin, Jan-Jong Hung

Supplementary Figure Legends

Suppl.Fig.1. USP24 increases expression of BRD-containing proteins. The interaction between USP24 and BRD-containing proteins was studied by yeast two-hybrid assay. The interaction motifs are shown (A) of the lysates of H1299 cells in which USP24 was knocked down were collected, and the protein levels were determined by Western blotting with the indicated antibodies (B).

Suppl.Fig.2. USP24 increases the protein stability of BRD-containing proteins. Knockdown of USP24 (A) and overexpression of GFP-USP24 (B) in lysates collected from U2OS cells with or without cycloheximide treatment were used to study the protein levels by Western blotting with the indicated antibodies. BRD7 was quantitated and the results were subjected to statistical analysis by t-test, p*<0.05, p**<0.01, after three independent experiments.

Suppl.Fig.3. USP24 interacts with BRD-containing proteins. MEF lysates were used to study the interaction between USP24, p300 and BRD7 by IP with anti-USP24 antibodies, following which Western blotting was carried out with the indicated antibodies (A). HA-BRG1 was expressed in MEFs for IP with anti-USP24 antibodies, following which Western blotting was performed with the indicated antibodies (B).

Suppl.Fig.4. The USP24 KD-mediated reduction in the HA-BRD level was reversed by MG132. Cells in which HA-BRD was overexpressed and USP24 was knocked down

in H1299 cells were treated with MG132, followed by Western blotting with the indicated antibodies.

Suppl.Fig.5. Comparison of the BRD sequences in various BRD-containing proteins. The sequences of BRDs from various BRD-containing proteins were aligned for comparison. The conserved lysine (K) residue is highlighted in red.

Suppl.Fig.6. Functional assay in U2OS cells overexpressing GFP-USP24 and HA-BRG1. GFP-USP24 and HA-BRG1 were expressed in U2OS cells, and their migratory ability was studied with a wound healing assay.

Suppl.Fig.7. Showing the additional repeated data.

A

Bromodomain -coantaining proteins	Name	Function	Bromodomain region	Protein region	USP24 region
BRD2	Bromodomain containing 2	Transcription regulator	91-163, 364-436 a.a.	367-669 a.a.	2078-2602 a.a.
BRD7	Bromodomain containing 7	Transcription regulator	148-218 a.a.	38-341 a.a.	2078-2602 a.a.
BRDT	Bromodomain, testis-specific	Chromatin remodeling factor	44-116, 287-359 a.a.	307-462 a.a.	2078-2602 a.a.

B

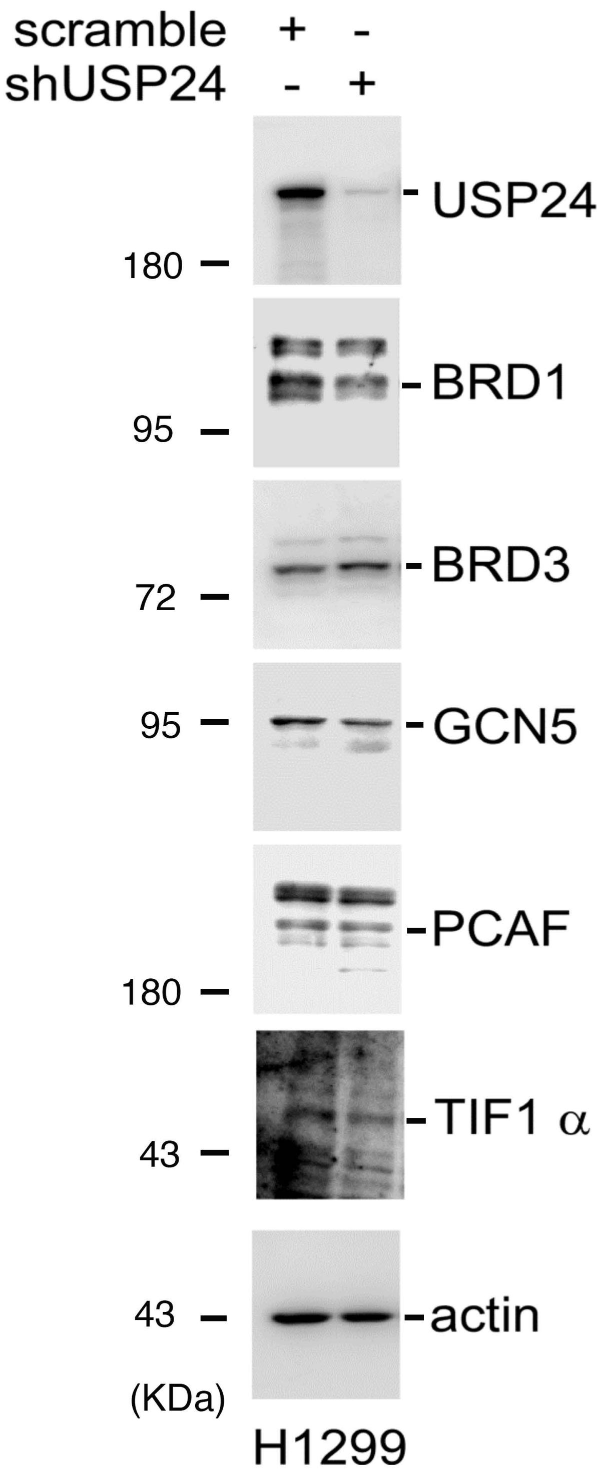

Suppl.Fig.1

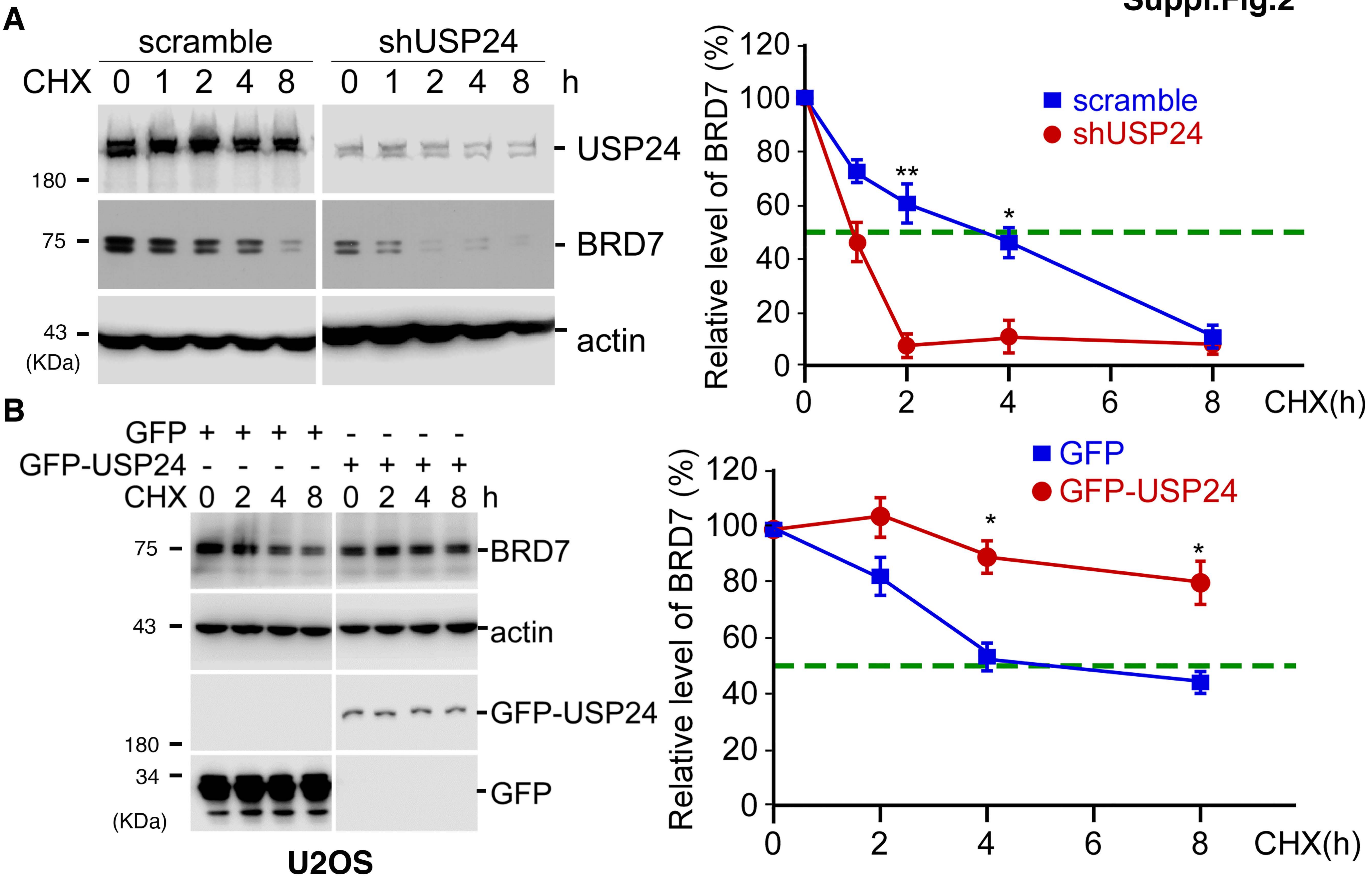
A
scramble
shUSP24
CHX 0 1 2 4 8 0 1 2 4 8 h
USP24
BRD7
actin
180
75
43
(KDa)
Relative level of BRD7 (%)
120
100
80
60
40
20
0
scramble
shUSP24
**
*
0 2 4 6 8 CHX(h)
B
GFP + + + + - - - -
GFP-USP24 - - - - + + + +
CHX 0 2 4 8 0 2 4 8 h
BRD7
actin
GFP-USP24
GFP
75
43
180
34
(KDa)
U2OS
GFP
GFP-USP24
*
*
0 2 4 6 8 CHX(h)

Supp.Fig.3

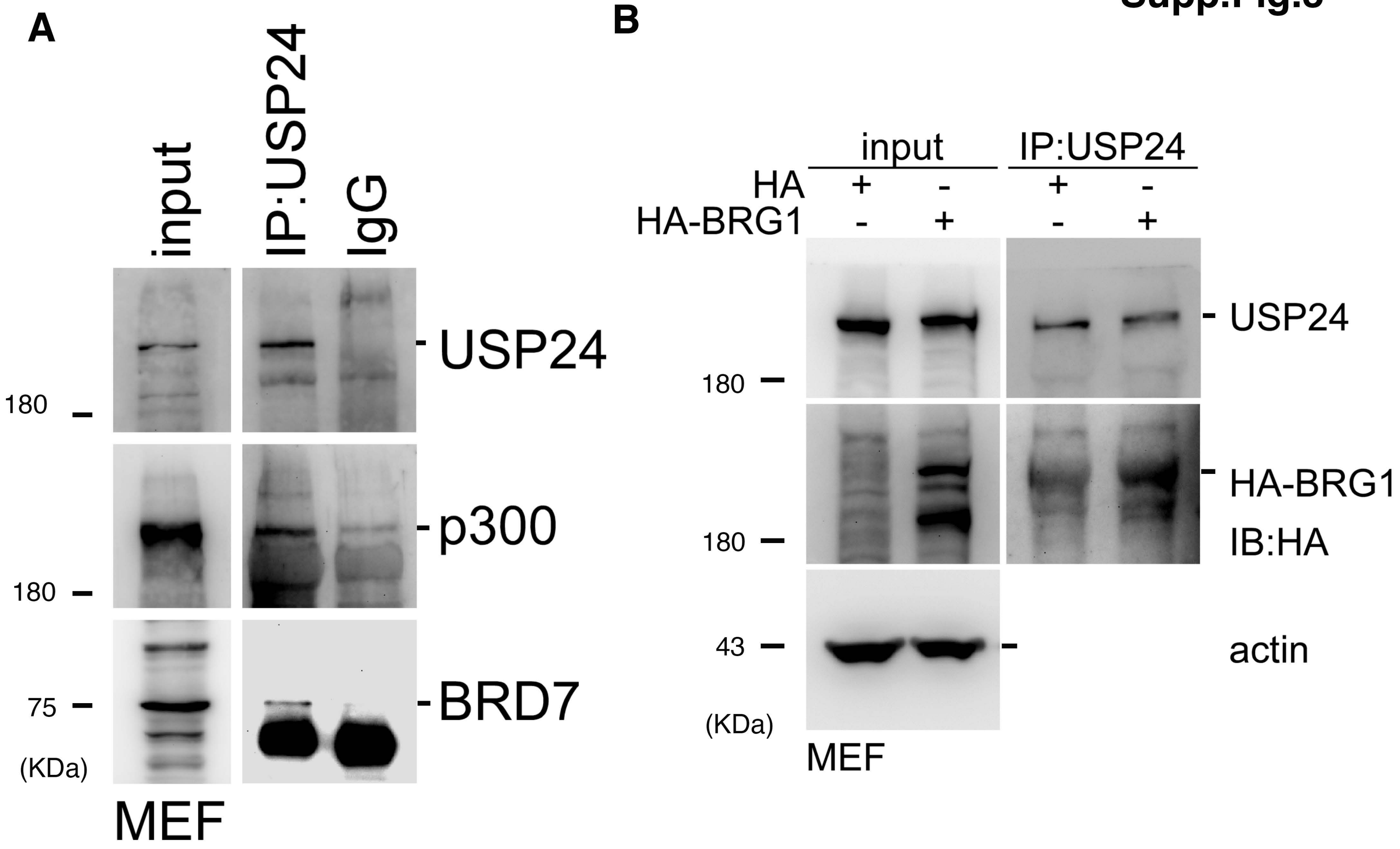
A
input
IP:USP24
IgG
USP24
p300
BRD7
180
180
75
(KDa)
MEF
B
input
IP:USP24
HA + - + -
HA-BRG1 - + - +
USP24
HA-BRG1
IB:HA
actin
180
180
43
(KDa)
MEF

Suppl.Fig.4

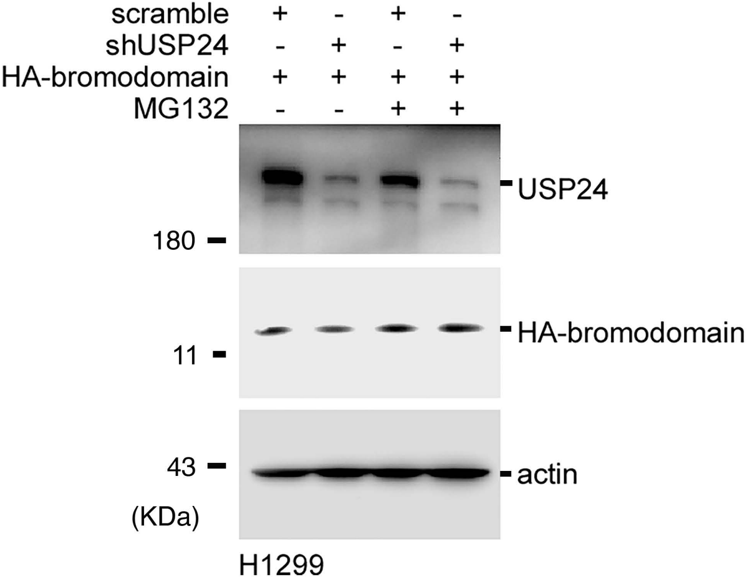
scramble + - + -
shUSP24 - + - +
HA-bromodomain + + + +
MG132 - - + +
USP24
180
HA-bromodomain
11
43
actin
(KDa)
H1299

Suppl.Fig.5

Bromodomain: 12K

GKLSEQLKHCNGILKELLSKKHAAYAWPFYKPVDASALGLHDYHDIIKHPMDLSTVKRKMENRDYRDAQEF AADVRLMFSNCYKYNPPDHDVVAMARKLQDVFEFRYAKMPD

	BRD2 2nd	GKLSEQLKHCNGILKELLSKKHAAYAWPFYKPVDASALGLHDYHDIIKHPMDLSTVKRKMENRDYRDAQEFAADVRLMFSNCYKYNPPDHDVVAMARKLQDVFEFRYAKMPD	364-436
*	BRD7	QRKDPSAFFSFPVTDFIAPGYSMIIKHPMDFSTMKEKIKNNDYQSIEELKDNFKLMCTNAMIYNKPETIYY	148-218
	BRG1	SGRQLSEVFIQLPSRKELPEYYELIRKPVDFKKIKERIRNHKYRSLNDLEKDVMLLCQNAQTFNLEGSLIY	1477-1547
**	p300	RQDPESLPFRQPVDPQLLGIPDYFDIVKSPMDLSTIKRKLDTGQYQEPWQYVDDIWLMFNNAWLYNRKTSRVY	1067-1139
**	BRDT 1st	WKHSFSWPFQRPVDAVKLQLPDYYTIIKNPMDLNTIKKRLENKYYAKASECIEDFNTMFSNCYLYNKPGDDIV	44-116
***	BRDT 2nd	KHFSYAWPFYNPVDVNALGLHNYYDVVKNPMDLGTIKEKMDNQEYKDAYKFAADVRLMFMNCYKYNPPDHEVV	287-359
**	BRD3 1st	WKHQFAWPFYQPVDAIKLNLPDYHKIIKNPMDMGTIKKRLENNYYWSASECMQDFNTMFTNCYIYNKPTDDIV	51-123
***	BRD3 2nd	KHAAYAWPFYKPVDAEALELHDYHDIIKHPMDLSTVKRKMDGREYPDAQGFAADVRLMFSNCYKYNPPDHEVV	326-398
**	BRD4 1st	WKHQFAWPFQQPVDAVKLNLPDYYKIIKTPMDMGTIKKRLENNYYWNAQECIQDFNTMFTNCYIYNKPGDDIV	75-147
***	BRD4 2nd	KHAAYAWPFYKPVDVEALGLHDYCDIIKHPMDMSTIKSKLEAREYRDAQEFGADVRLMFSNCYKYNPPDHEVV	368-440
**	CREBBP	RQDPESLPFRQPVDPQLLGIPDYFDIVKNPMDLSTIKRKLDTGQYQEPWQYVDDVWLMFNNAWLYNRKTSRVY	1103-1175
*	PCAF	KSHQSAWPFMEPVKRTEAP--GYYEVIRFPMDLKTMSERLKNRYYVSKKLFMADLQRVFTNCKEYNPPESEYY	740-810
*	GCN5L2	KSHPS-AWPFMEPVKKSEAP--DYYEVIRFPIDLKTMTERLRSRYYVTRKLFVADLQRVIANCREYNPPDSEYC	745-815
	SMARCA2	SGRQLSEVFIQLPSRKELPEYYELIRKPVDFKKIKERIRNHKYRSLGDLEKDVMLLCHNAQTFNLEGSQIY	1419-1489
**	FALZ	QAHKMAWPFLEPVDPN--DAPDYYGVIKEPMDLATMEERVQRRYYEKLTEFVADMTKIFDNCRYYNPSDSPFY	2944-3014
*	BRD1	QDKDPARIFAQPV--SLKEVPDYLDHIKHPMDFATMRKRLEAQGYKNLHEFEEDFDLIIDNCMKYNARDTVFY	579-649
*	ATAD2	AIDKRFRVFTKPVDPDEVP--DYVTVIKQPMDLSSVISKIDLHKYLTVKDYLRDIDLICSNALEYNPDRDPGD	1001-1071
	ZMYND11	RMKERAIDLNKKGKDNKHPMYRRLVHSAVDVPTIQEKVNEGKYRSYEEFKADAQLLLHNTVIFYGADSEQA	168-238
**	BRD9	QRKDPHGFFAFPV---TDAIAPG---YSMIIKHPMDFGTMKDKIVANEYKSVTEFKADFKLMCDNAMTYNRPDTVYY	153-223
*	BRPF3	QEKDPAHIFAEPVNLSEVP--DYLEFISKPMDFSTMRRKLESHLYRTLEEFEEDFNLIVTNCMKYNAKDTIFH	606-676
*	BRWD3 1st	LSLDFASPFAVPVDLSAYPL--YCTVVAYPTDLNTIRRRLENRFYRRISALMWEVRYIEHNARTFNEPDSPIV	1158-1228
*	KIAA1240	ATDKRFNIFSKPVDIEEVS--DYLEVIKEPMDLSTVITKIDKHNYLTAKDFLKDIDLICSNALEYNPDKDPGD	975-1045
*	BAZ2A	ESHDAAWPFLEPVNPRLV--SGYRRIIKNPMDFSTMRERLLRGGYTSSEEFAADALLVFDNCQTFNEDDSEVG	1810-1880
	BAF180 1st	QGRLLCELFIRAPKRRNQPDYYEVVSQPIDLMKIQQKLKMEEYDDVNLLTADFQLLFNNAKSYYKPDSPEY	64-134
*	BAF180 2nd	SGRLISELFQKLPSKVQYPDYYAIIKEPIDLKTIAQRIQNGSYKSIHAMAKDIDLLAKNAKTYNEPGSQVF	200-270
	BAF180 3rd	QGQLIAEPFYHLPSKKKYPDYYQQIKMPISLQQIRTKLKNQEYETLDHLECDLNLMFENAKRYNVPNSAIY	400-470
	BAF180 4th	SGRRLCDLFMVKPSKKDYPDYYKIILEPMDLKIIEHNIRNDKYAGEEGMIEDMKLMFRNARHYNEEGSQVY	538-608
	BAF180 5th	RGRRLSAIFLRLPSRSELPDYYLTIKKPMDMEKIRSHMMANKYQDIDSMVEDFVMMFNNACTYNEPESLIY	676-746

Suppl.Fig.6

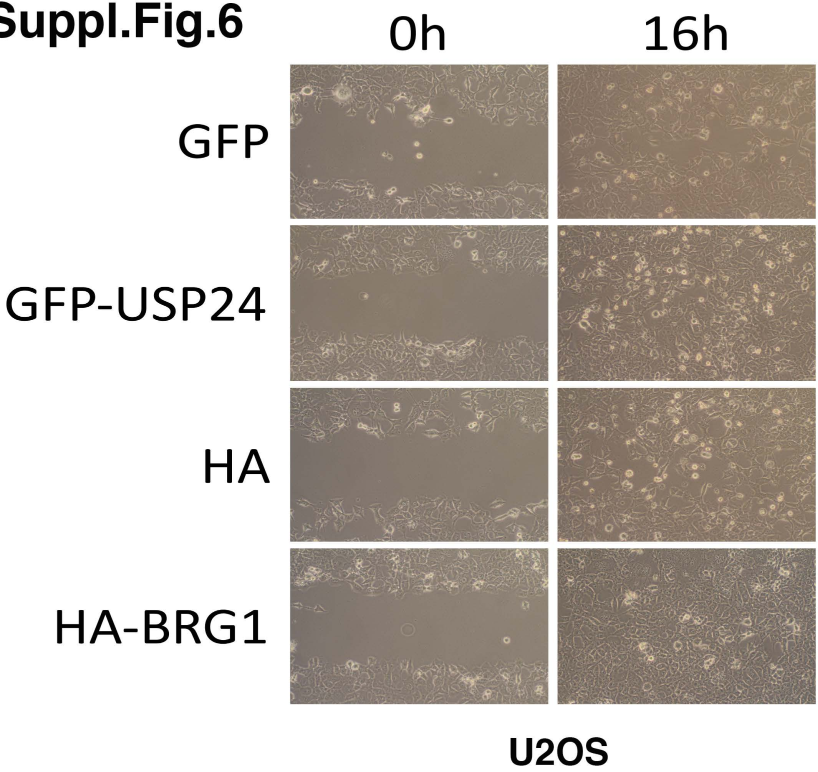

Suppl.Fig.7

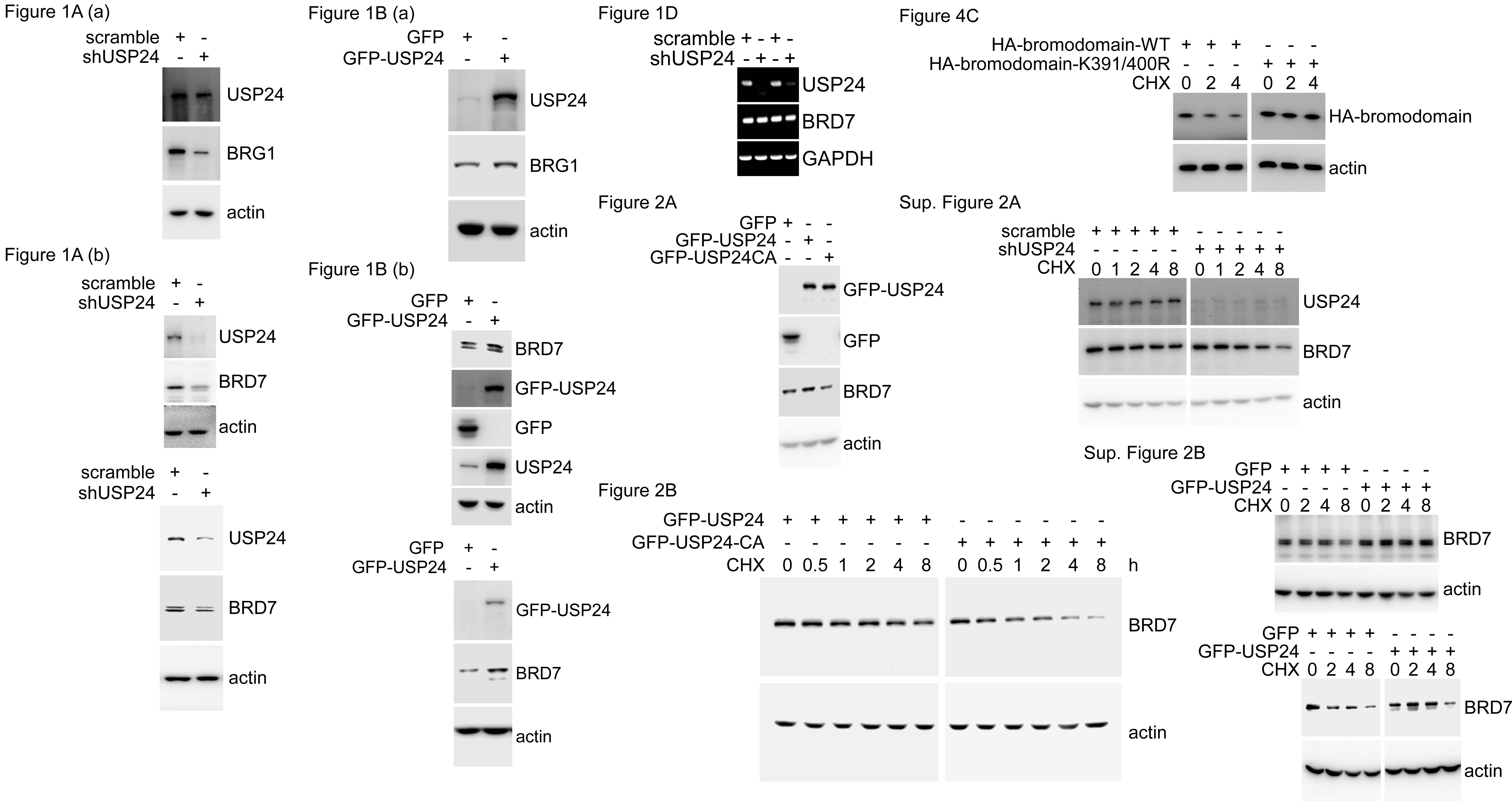
Figure 1A (a)
scramble + -
shUSP24 - +
USP24
BRG1
actin
Figure 1A (b)
scramble + -
shUSP24 - +
USP24
BRD7
actin
scramble + -
shUSP24 - +
USP24
BRD7
actin
Figure 1B (a)
GFP + -
GFP-USP24 - +
USP24
BRG1
actin
Figure 1B (b)
GFP + -
GFP-USP24 - +
BRD7
GFP-USP24
GFP
USP24
actin
GFP + -
GFP-USP24 - +
GFP-USP24
BRD7
actin
Figure 1D
scramble + - + -
shUSP24 - + - +
USP24
BRD7
GAPDH
Figure 2A
GFP + - -
GFP-USP24 - + -
GFP-USP24CA - - +
GFP-USP24
GFP
BRD7
actin
Figure 2B
GFP-USP24 + + + + + + - - - - - -
GFP-USP24-CA - - - - - - + + + + + +
CHX 0 0.5 1 2 4 8 0 0.5 1 2 4 8 h
BRD7
actin
Figure 4C
HA-bromodomain-WT + + + - - -
HA-bromodomain-K391/400R - - - + + +
CHX 0 2 4 0 2 4
HA-bromodomain
actin
Sup. Figure 2A
scramble + + + + + - - - - -
shUSP24 - - - - - + + + + +
CHX 0 1 2 4 8 0 1 2 4 8
USP24
BRD7
actin
Sup. Figure 2B
GFP + + + + - - - -
GFP-USP24 - - - - + + + +
CHX 0 2 4 8 0 2 4 8
BRD7
actin
GFP + + + + - - - -
GFP-USP24 - - - - + + + +
CHX 0 2 4 8 0 2 4 8
BRD7
actin
